# The Association Between Gastrointestinal Issues and Psychometric Scores in Children with Autism Spectrum Disorder, Developmental Delays, Down Syndrome, and Typical Development

**DOI:** 10.1007/s10803-024-06387-2

**Published:** 2024-05-13

**Authors:** Jennie Sotelo-Orozco, Irva Hertz-Picciotto

**Affiliations:** 1 Department of Public Health Sciences, University of California at Davis, Davis, CA, USA; 2 MIND Institute, School of Medicine, University of California Davis, Sacramento, CA 95817, USA

**Keywords:** Gastrointestinal issues, Autism spectrum disorder, Down syndrome, Psychometric, Constipation, Cognitive

## Abstract

Investigate the association between gastrointestinal (GI) issues and psychometric scores among children with developmental delays and typical development. We examined the association between GI issues and the Mullen Scale of Early Learning (MSEL), Vineland Adaptive Behavior Subscales (VABS), and Aberrant Behavior Checklist (ABC) scores from participants with autism spectrum disorder (ASD), Down syndrome (DS), other developmental delays (DD) and typical development (TD) from the CHildhood Autism Risk from Genetics and Environment (CHARGE) Study (*n* = 1603). Approximately 32% of children with ASD, 31% of children with DD, and 20% of children with DS reported at least one GI issue, compared to 7% of TD controls. Constipation was the most frequently reported symptom for the entire population, including controls. In general, GI issues correlated with poorer behavioral scores (decreased communication, daily living, socialization, and motor skills on the VABS, and increased irritability/agitation, lethargy/social withdrawal, stereotypic behavior, and hyperactivity/noncompliance on the ABC) among ASD cases. Analysis by sex indicated that GI issues also correlated with poorer cognitive scores (fine motor, receptive language, expressive language, and MSEL composite scores), and adaptive behavior (communication skills, daily living skills, motor, and VABS composite scores) among boys with DD, but not girls with DD—suggesting sex differences among DD cases. Even TD controls showed increased stereotypic behavior and social withdrawal in association with GI issues. However, GI issues were not correlated with impairments in psychometric scores among DS cases. Given that GI issues correlate with deficits in behavioral and cognitive scores, future studies should investigate the treatment of GI symptoms in children with ASD and DD.

## Introduction

Developmental disabilities are estimated to affect one in six children in the United States (Zablotsky et al., 2019). Examples of some common developmental disabilities include autism spectrum disorder (ASD), intellectual disability, learning disability, and Down syndrome (DS) among others. Developmental disabilities affect an individual’s physical, intellectual, and behavioral development, however, individuals with developmental delays often also have a high incidence of co-occurring conditions, including gastrointestinal (GI) issues (Chaidez et al., 2014; Restrepo et al., 2020; Reynolds et al., 2021). Chaidez et al. previously found that children with ASD were nearly 8 times more likely, and those with developmental delays (DD) were 4.5 times more likely, to have at least one frequent GI symptom compared to typically developed (TD) children (Chaidez et al., 2014). Furthermore, they found that ASD cases with frequent GI issues (abdominal pain, gaseousness, diarrhea, constipation, or pain on stooling) scored poorer on aberrant behavior subscales (ABC), including increased irritability, social withdrawal, stereotypy, and hyperactivity as compared to ASD cases without frequent GI issues. Similarly, among DD cases, they found diarrhea correlated with increased irritability, social withdrawal, and hyperactivity. Considering the brain and gut functions are closely integrated by a bidirectional communication called the gut-brain axis, the pain and discomfort caused by GI symptoms can understandably worsen behavior among children with developmental disabilities (Li & Zhou, 2016). Several other studies have found similar results, particularly in ASD. Ferguson et al. (Ferguson et al., 2019) found a relationship between GI problems and behavior among children with ASD, which varied by age group. They found that among young children with ASD (*n* = 200; 2–5 yrs.), those with aggression problems were more likely to experience nausea, while older children with ASD (*n* = 140; 6–18 yrs.) were more likely to experience adverse internalizing behavior (such as anxiety and withdrawn behavior) with constipation and stomachaches. Similarly, Mazurek et al. (Mazurek et al., 2013) reported a high correlation between GI issues (including constipation, abdominal pain, bloating, diarrhea, and/or nausea) and anxiety and sensory over-responsivity in children with ASD. Fulceri et al. (Fulceri et al., 2016) also found that children with ASD and GI issues had more anxiety problems, somatic complaints, and externalizing problems than ASD children without GI issues. Collectively these findings suggest GI issues may be correlated with some behavioral problems among children with ASD.

Unlike ASD, children with DS have unique features, such as neuromotor coordination impairments, and craniofacial and structural abnormalities (associated with an extra copy of chromosome 21) that can have direct and indirect consequences on gastrointestinal and feeding issues (Mizuno & Ueda, 2001). In a study conducted in a Down syndrome clinic among children less than 2 years of age, researchers found that 17% of DS patients had structural GI anomalies (anal stenosis, duodenal stenosis/atresia, imperforate anus, and Hirschsprung disease), and 57% of DS patients experienced some feeding problems, including constipation among 18% of participants, based on parent interview (Spahis & Wilson, 1999). Ravel et al. also found that some functional GI disorders (i.e. suction issues, swallowing, and chewing disorders), and gastrointestinal reflux were commonly reported among children and adults with DS (Ravel et al., 2020). Although GI issues are also common among individuals with DS, very little is known about how GI issues may affect behavioral and cognitive scores in children with DS, or children with other developmental delays apart from ASD.

As such, the present study sought to investigate the frequency of GI issues in the large ongoing case-control Childhood Autism Risk from Genetics and Environment (CHARGE) Study to examine the association between GI issues and psychometric scores among children with a broad range of developmental disabilities including ASD, DD, and DS as compared to typically developing controls. This study builds upon the work by Chaidez et al. (Chaidez et al., 2014), previously mentioned, which investigated GI issues in the CHARGE study and examined the association between GI issues and maladaptive scores on the Aberrant Behavior Checklist (ABC) subscales among CHARGE participants (*n* = 960) with ASD, DD, and TD. The present analysis expands on this previous work to include a larger sample size currently available (*n* = 1603), further distinguishes those with Down syndrome separately from those with other developmental delays, to investigate unique features associated with Trisomy 21 and includes additional outcomes: cognitive scores of the Mullen Scale of Early Learning (MSEL), and adaptive behavior on the Vineland Adaptive Behaviors Scales (VABS). Also, given certain developmental delays, including ASD, predominantly affect more boys than girls (Maenner et al., 2023), the present study also aimed to investigate if there were sex differences in the associations between GI issues and psychometric scores including maladaptive behavior on the ABC sub-scales, as well as scores on the MSEL and VABS.

## Methods

### Study Population

All children in the present study are from the Childhood Autism Risk from Genetics and Environment (CHARGE) Study (Hertz-Picciotto et al., 2006). The CHARGE Study is an ongoing population-based case-control study that began in 2003 and aims to uncover the environmental causes of autism and examine genetic factors and the interactions between genes and environment in the etiology of autism. It also seeks to understand phenotypic variability in children with ASD as well as DD. CHARGE eligible children met the following criteria: (a) aged between 24 and 60 months at recruitment, (b) living with a biological parent who speaks English or Spanish, (c) born in California, and e) residing in the study catchment areas. Participants were recruited from three strata: children with ASD, children with a developmental delay but not ASD (DDs), and children randomly sampled from the general population. Children with ASD and other DDs were recruited from the State of California Department of Developmental Services. The primary aim of the CHARGE study was to investigate ASD, and therefore, TD controls were matched for frequencies on age, sex, and broad geographic distribution of the autism cases. The community diagnosis of children was confirmed, or not confirmed, using a set of standardized clinical assessments. Diagnostic tools and algorithms to classify children into the final study groups of ASD, DDs, or TD groups are described elsewhere (Hertz-Picciotto et al., 2006). Only general population controls were placed in the TD group, in order to maintain population-based sampling. The CHARGE study was approved by the State of California Department of Developmental Services and the institutional review boards at the University of California, Davis. Informed consent was obtained from parents before participation and any collection of data.

**Fig. 1:**
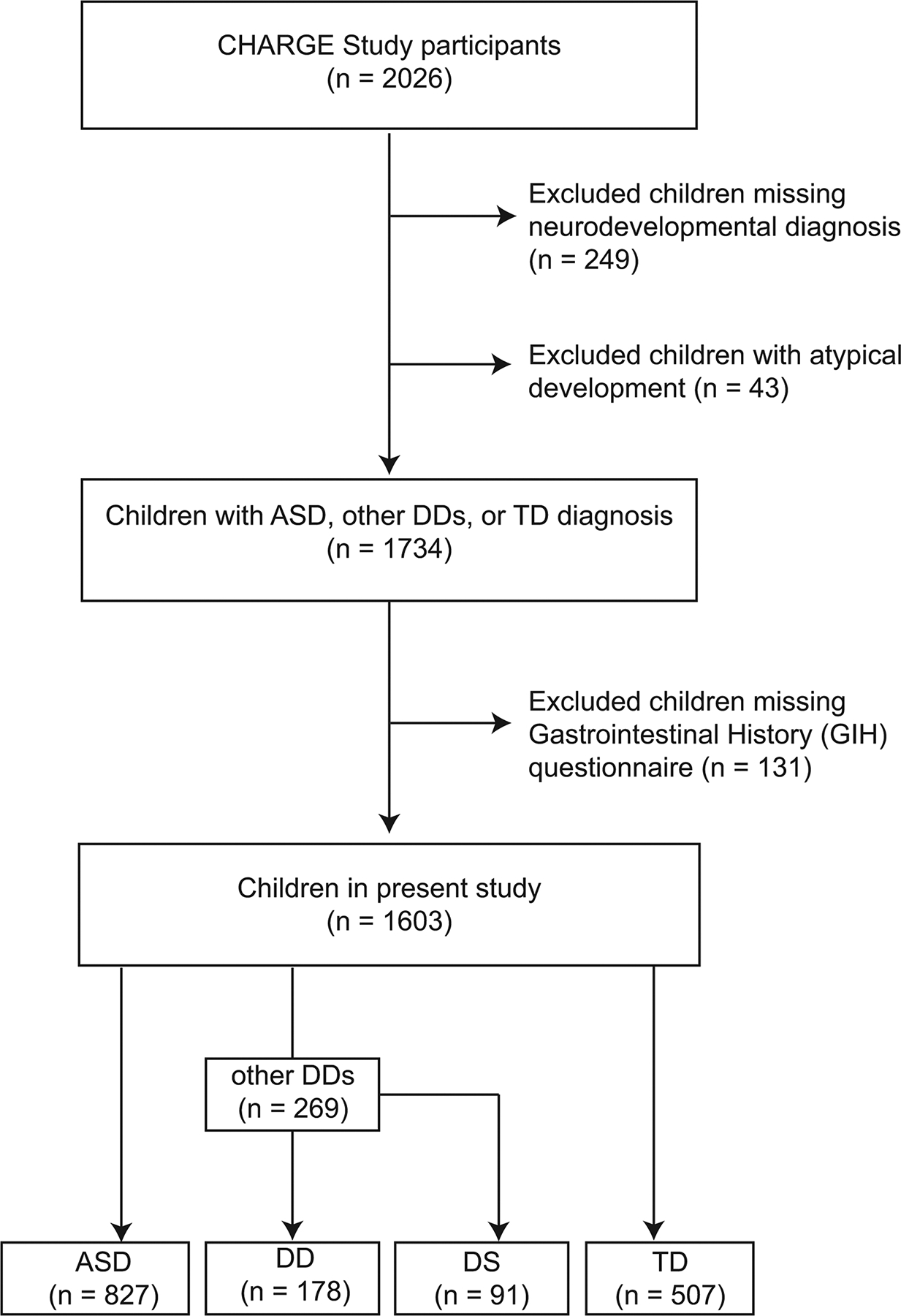
Flow chart of the study population

For this study, we considered a total of 2026 CHARGE study participants available in August 2023 for inclusion in this analysis. However, participants missing final neurodevelopmental diagnosis (*n* = 249), and children with atypical development (*n* = 43), who do not meet the criteria for ASD, DDs, or TD were excluded. Additionally, participants missing the Gastrointestinal History (GIH) questionnaire (*n* = 131) were also excluded. Therefore, a total of 1603 children were available for the present study including children with TD (*n* = 507), ASD (*n* = 827), and other DDs (*n* = 269)—children with other DDs were further subdivided into two groups: those with Down syndrome (DS, *n* = 91; based on parental report), and those with other types of developmental delays (DD, *n* = 178). Figure 1 presents the flowchart of the study population.

### Gastrointestinal Issues

The CHARGE Study collected a structured detailed parent-administered child GI history (GIH) questionnaire. Details about the CHARGE GIH have previously been published (Chaidez et al., 2014). For this study, we included GI symptoms of abdominal pain, gaseousness/bloating sensation, diarrhea, constipation, pain in stooling, vomiting, and/or difficulty swallowing. The frequency of GI issues present within the last 3 months was rated on a 3-point Likert scale (0= “never”, 1= “rarely or sometimes”, 2= “frequently or always”). Participants experiencing at least one GI symptom in the “frequently/always” range were categorized as having co-occurring GI symptoms (GI+), and those reporting GI symptoms in the ‘never” or “rarely/sometimes” ranges were classified as not having co-occurring GI issues (GI−). Among children with GI+, the number of GI symptoms reported was analyzed as a continuous variable.

### Psychometric Scores

Psychometric scores were assessed using the MSEL, VABS, and ABC. The MSEL is a standardized cognitive developmental test for children from birth to 68 months of age (Mullen, 1995) with four sub-scales: visual reception, fine motor, receptive language, and expressive language. Both the subscale scores and the composite scores were used in our analyses. MSEL scores are presented as the developmental quotient (DQ), meaning the ratio of the age-equivalent score to the chronological age. The VABS is a parent interview (Sparrow et al., 1984), which covers domains of socialization, daily living skills, motor skills, and communication. Composite scores were also determined, and similarly, to MSEL, VABS scores are also presented as the DQ. Additionally, parents completed the ABC (Aman et al., 1985) to rate inappropriate and maladaptive behaviors in their children. The ABC is one of the most widely used behavior rating scales and consists of 58 items, each scored on a four-point scale ranging from 0 (= not a problem) to 3 (= problem is severe in degree). The items are assigned to one of five subscales including irritability, agitation, and crying (15 items); lethargy, and social withdrawal (16 items); stereotypic behavior (7 items); hyperactivity, and noncompliance (16 items); and inappropriate speech (4 items). However, unlike the MSEL and VABS, where higher scores show improvements, higher ABC scores indicate poorer behavior (i.e. increased maladaptive behavior).

### Statistical Analysis

We computed univariate statistics and compared the population characteristics of the groups using the Chi-square test for categorical variables and the Kruskal-Wallis test for continuous variables. Multiple linear regression models were used to investigate the associations of GI issues (continuous) on each continuous outcome of the MSEL DQ and VABS DQ scores, stratified by neurodevelopmental diagnosis. To deal with the floor effect of ABC scores, negative binomial linear regression models were used to investigate the association between GI issues and ABC scores. Possible confounders for psychometric scores and GI issues were selected a priori based on a directed acyclic graph (DAG) (Fig. S1, Supplementary material). The online software tool DAGitty (http://www.dagitty.net/) was used to construct the DAG model (Textor et al., 2011). From the DAG, we identified a sufficient set of adjustment factors that would remove confounding and minimize bias in the estimated association between GI issues and psychometric scores. Final models were adjusted for parental homeownership, maternal prenatal vitamin use during the first month of pregnancy (based on previous evidence suggesting supplemental prenatal vitamin taken near conception significantly reduced the risk of autism (Schmidt et al., 2011)), and year of birth (as the distribution of diagnostic groups enrolled varied across years). As sensitivity analyses, we also ran the models limiting GI issues to only include constipation, as that was the most frequently reported GI symptom. Furthermore, as separate analyses, to examine effect modification of the GI associations with psychometric scores by sex, we performed additional analysis by refitting the same regression models (with the same covariate adjustment) but with an interaction term for sex and GI issues. We then examined stratum-specific estimates of the GI associations and their confidence limits and examined p-values for the interaction terms. In our analysis, p-values < 0.05 were considered significant. All analyses were performed using SAS software version 9.4 (SAS Institute Inc. 2016. SAS 9.4. Carny, NC) and R statistical language (R Foundation for Statistical Computing, Vienna, Austria) with RStudio.

## Results

Table 1 shows the study population characteristics. The population included 507 TD, 827 ASD, 178 DD, and 91 DS participants. In the CHARGE study, control subjects with TD were frequency matched to case subjects with ASD (4:1 male-to-female ratio) resulting in a skewed sex distribution and similar proportions of male subjects in ASD and TD groups; however, the developmentally delayed groups (DD and DS) were not sex-matched. Maternal education attainment was highest among parents with TD children, while parents of children with DD tended to have less formal education. As expected, mothers of children with DS were older than mothers of children with ASD, DD, and TD. Additionally, a greater proportion of mothers of children with TD had taken prenatal vitamins early in pregnancy compared to mothers of children with ASD, DD, or DS—which aligns with previous findings in the CHARGE study that maternal prenatal vitamin intake near conception significantly reduced the risk of having children with ASD and a trend towards decreased risk of having a child with developmental delays (Schmidt et al., 2011, 2012).

**Fig. 2:**
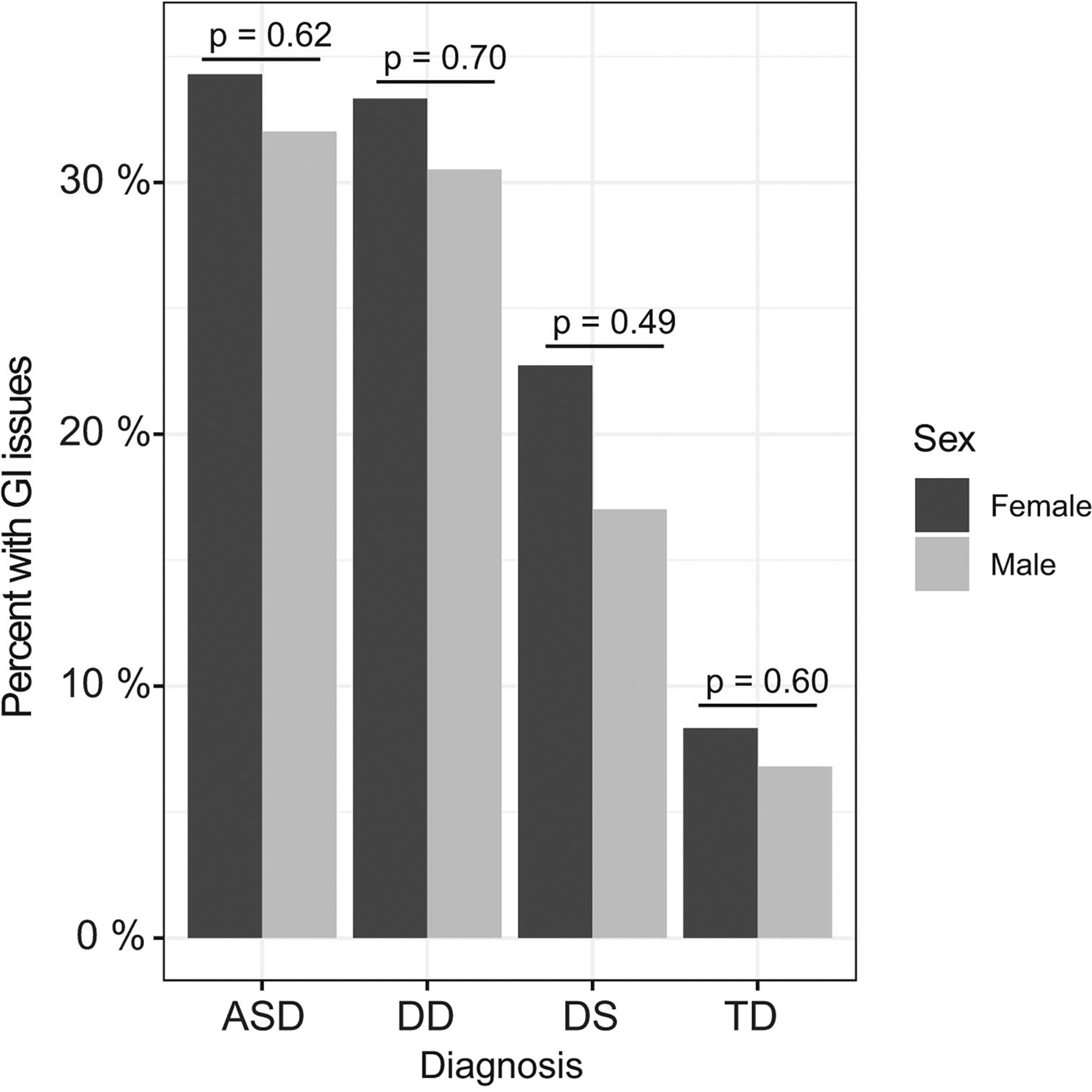
Distribution of males and females with GI issues (GI+) across the neurodevelopmental groups

Approximately 32% of children with ASD, 31% of children with DD, and 20% of children with DS reported commonly occurring GI issues (GI+), compared to only 7% of children with TD controls (Table 2). Constipation was the most frequently reported symptom among ASD and DD cases (affecting about 17% of these children), followed by diarrhea and gaseousness/bloating—although ASD cases had the highest frequencies for both of the latter GI issues, as well as pain on stooling. Similarly, among children with DS, constipation was also the most prevalent GI symptom affecting about 17% of DS cases. However, the other GI symptoms were rarely reported among DS cases (ranging from 0 to 2%, depending on the specific GI symptom). Even among children with TD, constipation was also the most prevalent GI symptom, but only affected about 4% of TD controls; the other GI issues were rarely reported among children with TD. Although there were clear differences in the presence of GI issues across neurodevelopmental groups, no significant difference was found in the frequency of GI issues between boys and girls within each neurodevelopmental group (i.e., boys with ASD vs. girls with ASD, etc.) (Fig. 2).

**Fig. 3:**
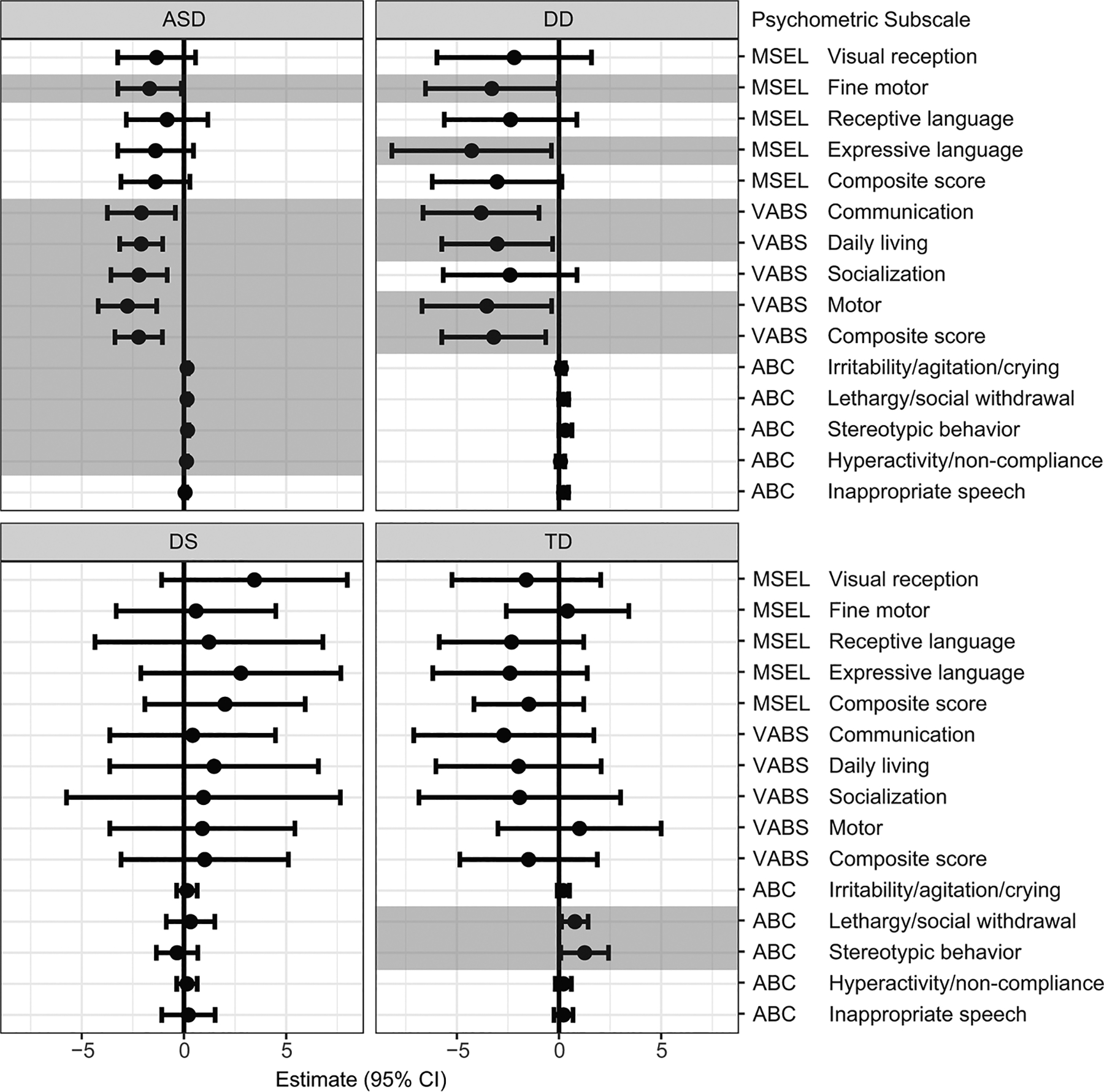
Results of regression models predicting psychometric scores based on GI issues, stratified by neurodevelopmental diagnosis. All models were adjusted for parental homeownership, maternal prenatal vitamin use during the first month of pregnancy, and the child’s year of birth. Significant associations (*p* < 0.05) are shown in shaded bar. The MSEL and VABS measure cognitive and adaptive behavior where higher scores indicate better performance, in contrast, ABC measures maladaptive behavior, therefore higher ABC scores indicate poorer behavior

Regression models were used to examine the association between GI issues and psychometric measurements for each diagnosis separately (Fig. 3). Table S1 (Supplementary material) presents a summary of the estimates (β) and 95% CI from the regression models. Among children with ASD, GI issues were associated with deficits in the MSEL subscale of fine motor [β = −1.68, 95% CI (−3.23, −0.12)], and VABS composite score and subscales (including communication [β = −2.08, 95% CI (−3.74, −0.42)], daily living [β = −2.09, 95% CI (−3.14, −1.04)], socialization [β = −2.20, 95% CI (−3.57, −0.82)], and motor skills [β = −2.76, 95% CI (−4.19, −1.33)], as well as increased maladaptive scores on the ABC (including irritability/agitation/crying [β = 0.16, 95% CI (0.10, 0.21)], lethargy/social withdrawal [β = 0.15, 95% CI (0.10, 0.21)], stereotypic behavior [β = 0.18, 95% CI (0.11, 0.24)], and hyperactivity/noncompliance [β = 0.12, 95% CI (0.08, 0.17)]). Similarly, among DD cases, GI issues were associated with deficits in MSEL subscales (fine motor [β = −3.29, 95%CI (−6.53, −0.04)], and expressive language [β = −4.27, 95%CI (−8.18, 0.37)]), and VABS composite score and subscales (including communication [β = −3.82, 95% CI −6.65, −0.98)], daily living [β = −3.03, 95% CI (−5.74, −0.32)], and motor skill [β = −3.53, 95% CI (−6.70, −0.36)]). Additionally, among TD cases GI issues were also associated with increased aberrant behavior subscales including lethargy/social withdrawal [β = 0.78, 95% CI (0.12, 1.43)], and stereotypic behavior [β = 1.25, 95% CI (0.08, 2.43)]. Surprisingly, among children with DS, GI issues were not associated with any composite or subscale score, and as seen in Figure 3, there was not even a trend in the direction of impaired scores or maladaptive behaviors. When restricting the analyses to only include constipation, similar results were observed across the groups, although the magnitude of the effect was greater compared to analyses that considered any GI issue (Table S2, Supplemental Material).

**Fig. 4:**
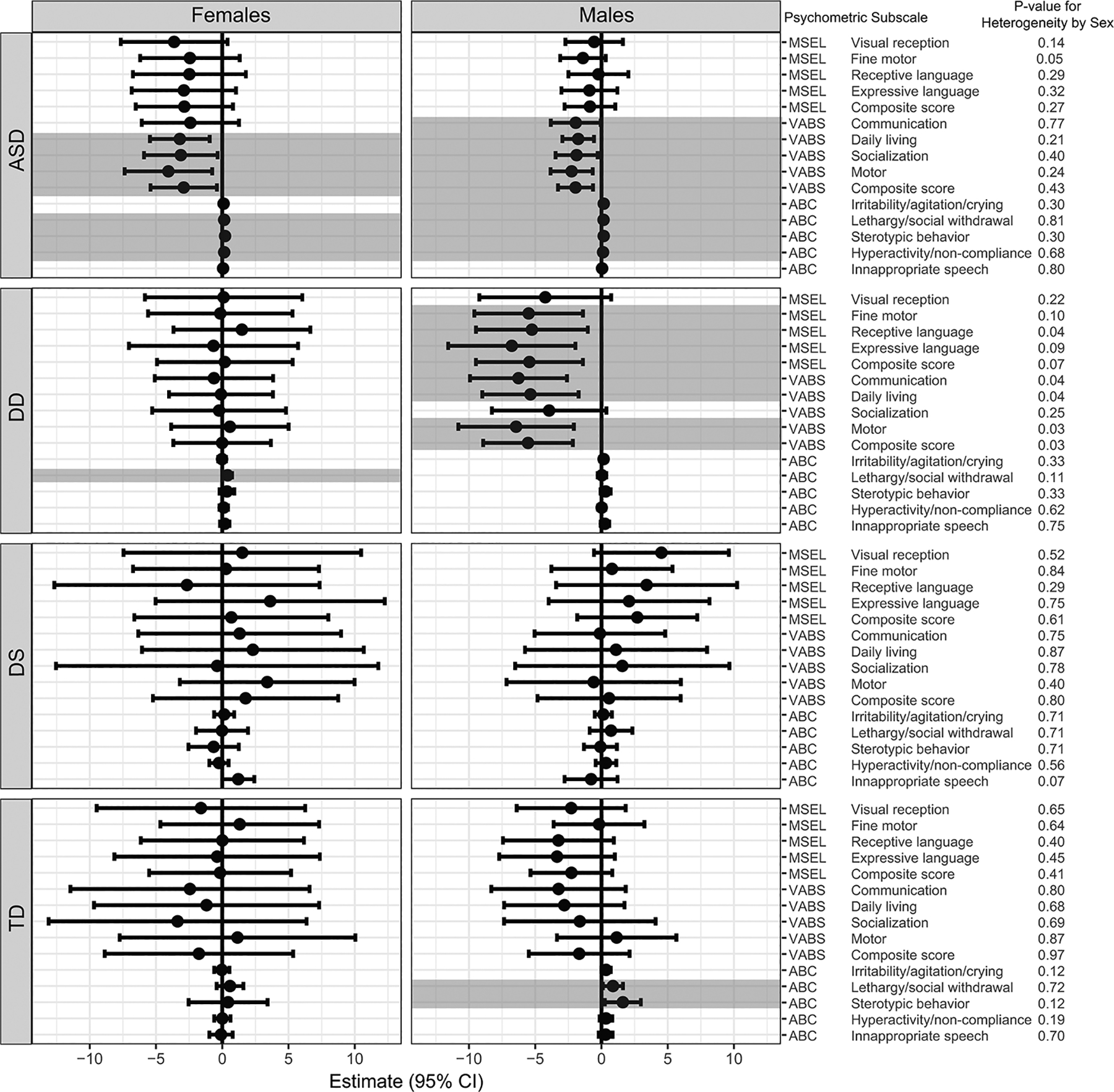
Results of regression models predicting psychometric scores based on GI issues, stratified by sex. All models were adjusted for parental homeownership, maternal prenatal vitamin use during the first month of pregnancy, and the child’s year of birth. Significant associations (*p* < 0.05) are shown in shaded bar. The p-value for heterogeneity from separate regression models is also presented. The MSEL and VABS measure cognitive and adaptive behavior where higher scores indicate better performance, in contrast, ABC measures maladaptive behavior, therefore higher ABC scores indicate poorer behavior

When our analyses were further stratified by sex, in general, we did not find significant sex differences among children with ASD, DS, or TD. However, there were significant sex differences in some psychometric scores predominantly among children with DD (Fig. 4). Among boys with DD, GI issues were significantly associated with deficits in MSEL composite score and MSEL subscales (including fine motor [β = −5.50, 95% CI (−9.22, −0.73)], receptive language [β = −5.25, 95% CI (−9.60, −1.39)], and expressive language [β = −6.77, 95% CI (−11.57, −1.97)]), and VABS composite score and subscales (communication [β = −6.26, 95% CI (−9.93, −2.60), daily living [β = −5.36, 95% CI (−9.00, −1.72)], and motor skill [β = −6.44, 95% CI (−10.80, −2.08)]). These associations were not found among girls with DD, however, girls with DD had increased lethargy/social withdrawal on the ABC in association with GI issues [β = 0.40, 95% CI (0.07, 0.74)]. Table S3 (Supplementary material) presents a summary of the estimates (β) and 95% CI from regression models for psychometric scores and GI issues for each group, stratified by sex.

## Discussion

Results from this study support the evidence that GI issues are more commonly reported among children with developmental delays compared to typically developing children (Chaidez et al., 2014; Leader et al., 2022; Sullivan, 2008). In this study, we found that 32% of children with ASD, 31% of children with DD, and 20% of children with DS in our study population reported commonly occurring GI issues, compared to only 7% of children with TD controls. Additionally, among the GI symptoms investigated (abdominal pain, gaseousness/bloating sensation, diarrhea, constipation, pain in stooling, vomiting, and/or difficulty swallowing), constipation was the most frequently reported symptom, irrespective of neurodevelopmental diagnosis—affecting approximately 16% of children with ASD, DD, and DS equally, but only 4% of children with TD. Furthermore, among ASD and DD cases, other GI symptoms, such as diarrhea and gaseousness/bloating were also commonly reported. However, these other GI issues were rarely reported among DS cases and TD controls.

Our results align with previous studies which have also found constipation is a frequently reported GI symptom among children with ASD (Bresnahan et al., 2015; Leader et al., 2022; Restrepo et al., 2020; Reynolds et al., 2021). Chronic constipation in ASD may be related to limited food preferences commonly observed among children with ASD. For example, one study found that food selectivity in ASD was indirectly associated with constipation (Harris et al., 2021). Unlike ASD however, in children with DS, constipation may be related to lower muscle tone (hypotonia) common in kids with DS, making bowel movements more difficult to pass (Dey et al., 2013). Additionally, individuals with DS are also prone to other conditions that can exacerbate constipation including hypothyroidism, Hirschsprung disease, and celiac disease (Marder & Jackson, 2018). A retrospective cross-sectional study of 1,207 patients with DS (between 0 and 31 + years of age), also found that constipation was the most prevalent GI symptom reported during a 10-year follow-up period (Bermudez et al., 2019). Additionally, in a cross-sectional survey study of children and adolescents with DS (*n* = 114; ages 4–18 years), researchers also found constipation affected 36% of DS cases (Ciciora et al., 2023). Overall, GI issues, and constipation in particular, are significantly more prevalent among individuals with developmental disabilities compared to neurotypical controls.

When we investigated the association between GI issues and psychometric scores, we found that among ASD cases the presence of GI issues broadly correlated with deficits in fine motor skills on the MSEL, and poorer adaptive skills (decreased communication, daily living skills, socialization, and motor skills and composite scores on the VABS), as well as increased maladaptive behavior (irritability/agitation/crying, lethargy/social withdrawal, stereotypic behavior, and hyperactivity/noncompliance on the ABC)—emphasizing a pattern of behavioral issues and interestingly also motor deficits associated with GI issues in ASD cases. Maenner et al. previously also found an association between GI issues and delayed motor milestones in a population-based study of children with ASD (Maenner et al., 2012). Furthermore, similar results were also observed when we limited the analyses to only include constipation, although the magnitude of the effect was greater for constipation, suggesting chronic constipation increases symptom severity. Additionally, when we further stratified our analysis by sex, in general, we did not find differences between boys and girls with ASD in relation to GI issues—as both sexes largely showed deficits in behavioral scores, but not cognitive scores, in association with GI issues. Our results line up with previous findings in the CHARGE Study (Chaidez et al., 2014) which likewise found that GI issues correlated with four of the five subscales for the ABC among children with ASD; although MSEL and VABS scores were not previously investigated by Chaidez et al. Similarly, Restrepo et al. also found GI symptoms were associated with increased self-injurious behaviors, somatic complaints, reduced sleep duration, and increased parasomnias among children with ASD and also did not find significant differences based on sex among ASD cases (Restrepo et al., 2020). Prosperi and colleagues likewise did not find a significant difference in GI issues comparing boys and girls with ASD but did find GI issues correlated with internalizing and externalizing problem scores of the Child Behavior Checklist questionnaire (Prosperi et al., 2017). Babinska et al. also found that the frequency of GI symptoms weakly correlated with the severity of the core symptoms of ASD based on Autism Diagnostic Interview-Revised (ADI-R) scores among children (ages 2–17 years) with ASD (Babinska et al., 2020). However, they did find GI issues were more common in girls with ASD than in boys with ASD (70.6% vs. 44.5%). Additionally, Mazefsky et al., which investigated older participants (7 to 19 years old), also found that girls with ASD had a higher frequency of GI issues compared to boys, and found GI issues were associated with higher levels of affective problems among ASD participants, after adjusting for sex and age (Mazefsky et al., 2014). While there may be some contradictory evidence regarding whether the prevalence of GI issues differs by sex among ASD cases, it is also possible that the frequency of GI symptoms may change with age in a sex-dependent manner and requires further investigation. Nonetheless, collectively these studies indicate GI issues, and constipation in particular, are associated with increased symptom severity.

Similar, to ASD cases, we also found that GI issues correlated with deficits in behavioral scores among children with DD. However, in contrast to ASD, GI issues were also correlated with poorer cognitive scores among DD cases, and when we stratified our analysis by sex, GI issues strongly correlated with broad impairments in MSEL cognitive subscales and deficits in VABS adaptive behavior subscales among boys with DD, but not among girls with DD—suggesting possible sex differences in psychometric subscales associated with DD. Girls with DD, however, did show increased lethargy/social withdrawal in association with GI issues. Interestingly, we also found GI issues correlated with increased lethargy/social withdrawal and stereotypic behavior even among TD cases. Similarly, in the large case-control Study to Explore Early Development (SEED), Reynolds et al. also found that children from all three diagnostic groups (including ASD, other developmental delays, and the general population) with GI symptoms had more aggression, anxiety, attention, and sleep concerns that children without GI symptoms (Reynolds et al., 2021)—suggesting that irrespective of neurodevelopmental diagnosis GI issues are associated with poorer behavioral/cognitive scores. Our findings were similar, with the exception that in DS children—who were not separated out in the Reynolds Study—GI issues were not associated with any psychometric scores, nor did we find significant sex differences in GI effects in this group. Surprisingly, several MSEL and VABS subscales positively correlated with GI issues among DS cases, although none reached statistical significance. Nevertheless, it is difficult to discern if this null result may be due to the relatively small number of DS cases reporting GI issues in our study, and therefore further research is necessary in a larger DS population. Apart from DS cases, our results indicate that GI issues negatively affect varying aspects of behavioral and cognitive scores differently across neurodevelopmental diagnosis, and in DD children by sex.

The present study adds to the existing literature by investigating a comprehensive set of psychometric scores in association with GI issues and further examines possible sex differences in these associations in a large population across a broad range of neurodevelopmental disabilities. Strengths in our study were that controls and cases alike were recruited based on the same eligibility criteria and that ASD and developmental delay diagnoses were clinically confirmed (as was TD) by psychometricians who had demonstrated research reliability on the instruments they administered. All the psychometric instruments used are established, standardized, and normed for the ages of the CHARGE study population. As such, the behavioral/ neurodevelopmental instruments in this study have enabled us to examine the GI issues with well-characterized behavioral and developmental phenotypes based on specific functional domains in children with developmental disabilities and to compare with controls selected from the same population and confirmed to have no developmental diagnosis (i.e., the TDs). Additionally, the children were all within a relatively narrow age range. Nonetheless, a limitation of our study is that GI symptoms were parent-reported utilizing the GI history (GIH) questionnaire developed for CHARGE with a reduced number of questions to limit the burden on participating families and aims to capture the most common symptoms exhibited by ASD, but which has not been validated. Additionally, GI symptoms were not further investigated with a medical evaluation. While other studies have also utilized parent-reported questionnaires to assess GI issues among children with developmental disabilities (Bresnahan et al., 2015; Restrepo et al., 2020), it is recommended that the evaluation of GI issues be ideally accomplished by a combination of medical history and physical examination (Buie et al., 2010). Future studies could utilize standardized GI assessment instruments, such as the Rome IV Diagnostic Questionnaire to screen for functional gastrointestinal disorders (Drossman, 2016; Hyman et al., 2006) to enable comparisons across studies when formal GI assessments are not feasible. Another limitation of this study is the smaller sample size for the DD and DS groups, as well as the fewer girls than boys included in our ASD and TD groups, which likely reduced our statistical power for these analyses.

Our results provide evidence that GI issues negatively affect behavioral scores among children with ASD. Additionally, we also found GI issues were associated with deficits in both cognitive and adaptive skills along with greater maladaptive behavior among boys, but not girls, with DD—suggesting a sex difference among DD cases. Given some GI symptoms can be treated, clinicians and parents of children with ASD and DD should look for signs of GI issues, particularly where behavioral problems have been observed. Additionally, a larger study among children with DS is necessary to corroborate the null results for GI issues in association with psychometric scores, considering children with DS also experience a high frequency of GI issues.

## Supplementary Material

Supplementary Material

## Figures and Tables

**Table 1:** Characteristics of study participants

Characteristics^1^	TD (*N* = 507)	ASD (*N* = 827)	DD (*N* = 178)	DS (*N*=91)	*P*-value^2^
	Freq (%)	Freq (%)	Freq (%)	Freq (%)	
**Child’s sex**					< 0.0001
Female	96 (18.9%)	140 (16.9%)	60 (33.7%)	44 (48.4%)	
Male	411 (81.1%)	687 (83.1%)	118 (66.3%)	47 (51.6%)	
**Child’s year of birth**					< 0.001
1998–2002	120 (23.7%)	314 (38.0%)	47 (26.4%)	7 (7.69%)	
2003–2006	189 (37.3%)	208 (25.2%)	67 (37.6%)	28 (30.8%)	
2007–2010	130 (25.6%)	131 (15.8%)	40 (22.5%)	34 (37.4%)	
2011–2014	52 (10.3%)	141 (17.1%)	18 (10.1%)	16 (17.6%)	
2015–2019	16 (3.2%)	33 (4.0%)	6 (3.4%)	6 (6.6%)	
**Child’s age** (months)					< 0.0001
Mean (SD)	44.0 (9.7)	46.0 (9.6)	47.5 (8.7)	45.4 (9.5)	
**Child’s race/ethnicity**					0.0002
Hispanic	102 (20.1%)	174 (21.1%)	33 (18.5%)	11 (12.1%)	
Non-Hispanic, Non-White	136 (26.9%)	259 (31.4%)	77 (43.7%)	40 (44.4%)	
White	266 (52.7%)	392 (47.5%)	66 (37.5%)	39 (43.3%)	
**Maternal education**					< 0.0001
High school graduate or less	64 (12.6%)	134 (16.2%)	57 (32.0%)	19 (20.9%)	
Some college, technical, vocational, or associate degree	170 (33.5%)	340 (41.2%)	67 (37.6%)	34 (37.4%)	
Bachelor’s degree	187 (36.9%)	233 (28.2%)	45 (25.3%)	29 (31.9%)	
Graduate or professional degree	86 (17.0%)	119 (14.4%)	9 (5.1%)	9 (9.9%)	
**Homeownership**					< 0.0001
No	121 (24.3%)	285 (36.4%)	75 (43.6%)	31 (36.9%)	
Yes	377 (75.7%)	498 (63.6%)	97 (56.4%)	53 (63.1%)	
**Maternal age at child’s birth** (years)					< 0.0001
Mean (SD)	30.8 (5.57)	30.5 (5.56)	29.2 (6.31)	34.5 (6.04)	
**Prenatal vitamin use in the first month of pregnancy**					0.03
No	162 (33.5%)	313 (42.0%)	64 (39.3%)	33 (41.2%)	
Yes	322 (66.5%)	433 (58.0%)	99 (60.7%)	47 (58.8%)	

1 Missing (n): race/ethnicity (8), maternal education (< 5), payer at delivery (25), homeownership (66), prenatal vitamin use (129)

2 P-value from Pearson’s chi-square test for categorical variables and the Kruskal-Wallis test for continuous variables

**Table 2:** Frequency of GI symptoms experienced by participants across neurodevelopmental groups

*N* (%)	TD	ASD	DD	DS	*p*-value^2^
GI+	36 (7.1%)	268 (32.4%)	56 (31.5%)	18 (19.8%)	< 0.0001
Specific GI symptoms^1^					
Constipation	19 (3.8%)	136 (16.6%)	30 (16.9%)	15 (16.7%)	< 0.0001
Diarrhea	7 (1.4%)	96 (11.8%)	12 (6.9%)	< 5 (--%)	< 0.0001
Gaseousness/bloating	10 (2.0%)	78 (9.9%)	11 (6.4%)	< 5 (--%)	< 0.0001
Pain on stooling	8 (1.6%)	55 (6.9%)	10 (5.8%)	0 (0%)	< 0.0001
Abdominal pain	7 (1.4%)	39 (5.0%)	8 (4.7%)	< 5 (--%)	< 0.0001
Difficulty swallowing	< 5 (--%)	29 (3.6%)	9 (5.2%)	< 5 (--%)	< 0.0001
Vomiting	< 5(--%)	18 (2.2%)	10 (5.8%)	< 5 (--%)	< 0.0001

1 Missing (n) for GI symptoms: abdominal pain (68), gaseousness (58), diarrhea (19), constipation (19), pain on stooling (35), vomiting (15), difficulty swallowing (40)

2 P-value from Pearson’s chi-square test; Fisher’s exact test used where applicable

## Data Availability

The datasets generated during and/or analyzed during the current study are available from the corresponding author upon reasonable request.
