# Supplementary material for: The Association Between Gastrointestinal Issues and Psychometric Scores in Children with Autism Spectrum Disorder, Developmental Delays, Down Syndrome, and Typical Development
Source: J Autism Dev Disord. 2024 May 13;55(7):2452–62. doi: 10.1007/s10803-024-06387-2 (PMC11557733; doi:10.1007/s10803-024-06387-2)
Supplement: Supplementary file 1 — Supplementary Material 1 [file 10803_2024_6387_MOESM1_ESM.docx]

**Supplementary Material**

**Supplementary Tables**

- **Table S1.** Beta estimates (95% CI) from regression models investigating the association between GI issues and psychometric scores.
- **Table S2.** Beta estimates (95% CI) from regression models investigating the association between constipation and psychometric scores.
- **Table S3.** Beta estimates (95% CI) from regression models investigating the association between GI issues and psychometric scores, stratified by sex.

**Supplementary Figures**

- **Fig S1.** Directed acyclic graph (DAG)

**Supplementary Table 1:** Results of regression models predicting psychometric scores based on GI issues, stratified by neurodevelopmental diagnosis. Models are adjusted for parental homeownership, maternal prenatal vitamin intake, and child’s year of birth. Significant associations (p< 0.05) are shown in bold.

| Dx | Test | Subscale | Estimate | 95% CI | | p-value |
| --- | --- | --- | --- | --- | --- | --- |
|  |  |  |  | **Lower**  **Bound** | **Upper**  **Bound** |  |
| ASD | MSEL | Visual reception | -1.34 | -3.24 | 0.57 | 0.17 |
|  |  | **Fine motor** | **-1.68** | **-3.23** | **-0.12** | **0.03** |
|  |  | Receptive language | -0.82 | -2.82 | 1.17 | 0.42 |
|  |  | Expressive language | -1.39 | -3.24 | 0.47 | 0.14 |
|  |  | Composite score | -1.40 | -3.08 | 0.29 | 0.10 |
|  | VABS | **Communication** | **-2.08** | **-3.74** | **-0.42** | **0.01** |
|  |  | **Daily living** | **-2.09** | **-3.14** | **-1.04** | **< 0.001** |
|  |  | **Socialization** | **-2.20** | **-3.57** | **-0.82** | **< 0.001** |
|  |  | **Motor** | **-2.76** | **-4.19** | **-1.33** | **< 0.001** |
|  |  | **Composite score** | **-2.22** | **-3.38** | **-1.06** | **< 0.001** |
|  | ABC | **Irritability, agitation, crying** | **0.16** | **0.10** | **0.21** | **<0.0001** |
|  |  | **Lethargy, social withdrawal** | **0.15** | **0.10** | **0.21** | **<0.0001** |
|  |  | **Stereotypic behavior** | **0.18** | **0.11** | **0.24** | **<0.0001** |
|  |  | **Hyperactivity, non-compliance** | **0.12** | **0.08** | **0.17** | **<0.0001** |
|  |  | Inappropriate speech | 0.06 | -0.02 | 0.14 | 0.17 |
| DD | MSEL | Visual reception | -2.19 | -5.97 | 1.59 | 0.25 |
|  |  | **Fine motor** | **-3.29** | **-6.53** | **-0.04** | **0.047** |
|  |  | Receptive language | -2.37 | -5.62 | 0.88 | 0.15 |
|  |  | **Expressive language** | **-4.27** | **-8.18** | **-0.37** | **0.03** |
|  |  | Composite score | -3.03 | -6.20 | 0.14 | 0.06 |
|  | VABS | **Communication** | **-3.82** | **-6.65** | **-0.98** | **0.01** |
|  |  | **Daily living** | **-3.03** | **-5.74** | **-0.32** | **0.03** |
|  |  | Socialization | -2.39 | -5.67 | 0.88 | 0.15 |
|  |  | **Motor** | **-3.53** | **-6.70** | **-0.36** | **0.03** |
|  |  | **Composite score** | **-3.19** | **-5.74** | **-0.65** | **0.01** |
|  | ABC | Irritability, agitation, crying | 0.11 | -0.08 | 0.31 | 0.25 |
|  |  | Lethargy, social withdrawal | 0.23 | -0.01 | 0.47 | 0.06 |
|  |  | Stereotypic behavior | 0.32 | -0.01 | 0.64 | 0.06 |
|  |  | Hyperactivity, non-compliance | 0.07 | -0.14 | 0.28 | 0.51 |
|  |  | Inappropriate speech | 0.22 | -0.01 | 0.46 | 0.06 |
| DS | MSEL | Visual reception | 3.45 | -1.09 | 7.99 | 0.13 |
|  |  | Fine motor | 0.59 | -3.31 | 4.50 | 0.76 |
|  |  | Receptive language | 1.22 | -4.35 | 6.80 | 0.66 |
|  |  | Expressive language | 2.78 | -2.11 | 7.67 | 0.26 |
|  |  | Composite score | 2.01 | -1.91 | 5.93 | 0.31 |
|  | VABS | Communication | 0.43 | -3.63 | 4.48 | 0.83 |
|  |  | Daily living | 1.47 | -3.63 | 6.58 | 0.57 |
|  |  | Socialization | 0.95 | -5.74 | 7.65 | 0.78 |
|  |  | Motor | 0.90 | -3.63 | 5.43 | 0.69 |
|  |  | Composite score | 1.02 | -3.07 | 5.11 | 0.62 |
|  | ABC | Irritability, agitation, crying | 0.15 | -0.36 | 0.65 | 0.56 |
|  |  | Lethargy, social withdrawal | 0.32 | -0.87 | 1.51 | 0.60 |
|  |  | Stereotypic behavior | -0.34 | -1.35 | 0.68 | 0.52 |
|  |  | Hyperactivity, non-compliance | 0.15 | -0.35 | 0.65 | 0.56 |
|  |  | Inappropriate speech | 0.22 | -1.09 | 1.52 | 0.74 |
| TD | MSEL | Visual reception | -1.60 | -5.24 | 2.04 | 0.39 |
|  |  | Fine motor | 0.42 | -2.58 | 3.41 | 0.78 |
|  |  | Receptive language | -2.32 | -5.86 | 1.21 | 0.20 |
|  |  | Expressive language | -2.40 | -6.18 | 1.38 | 0.21 |
|  |  | Composite score | -1.48 | -4.16 | 1.21 | 0.28 |
|  | VABS | Communication | -2.70 | -7.10 | 1.70 | 0.23 |
|  |  | Daily living | -1.98 | -6.02 | 2.06 | 0.34 |
|  |  | Socialization | -1.92 | -6.86 | 3.01 | 0.44 |
|  |  | Motor | 1.00 | -2.99 | 5.00 | 0.62 |
|  |  | Composite score | -1.49 | -4.85 | 1.88 | 0.39 |
|  | ABC | Irritability, agitation, crying | 0.21 | -0.09 | 0.51 | 0.18 |
|  |  | **Lethargy, social withdrawal** | **0.78** | **0.12** | **1.43** | **0.02** |
|  |  | **Stereotypic behavior** | **1.25** | **0.08** | **2.43** | **0.04** |
|  |  | Hyperactivity, non-compliance | 0.21 | -0.18 | 0.61 | 0.29 |
|  |  | Inappropriate speech | 0.22 | -0.25 | 0.68 | 0.36 |

**Supplementary Table 2**: Results of regression models predicting psychometric scores limited to constipation only, stratified by neurodevelopmental diagnosis. Models are adjusted for parental homeownership, maternal prenatal vitamin intake, and child’s year of birth. Significant associations (p< 0.05) are shown in bold.

| Dx | Test | Subscale | Estimate | 95% CI | | p-value |
| --- | --- | --- | --- | --- | --- | --- |
|  |  |  |  | **Lower**  **Bound** | **Upper**  **Bound** |  |
| ASD | MSEL | Visual reception | -2.36 | -7.40 | 2.68 | 0.36 |
|  |  | **Fine motor** | **-5.14** | **-9.18** | **-1.10** | **0.01** |
|  |  | Receptive language | -2.28 | -7.53 | 2.97 | 0.39 |
|  |  | Expressive language | -3.84 | -8.73 | 1.05 | 0.12 |
|  |  | Composite score | -3.50 | -7.91 | 0.90 | 0.12 |
|  | VABS | Communication | -3.92 | -8.25 | 0.41 | 0.08 |
|  |  | **Daily living** | **-3.50** | **-6.29** | **-0.71** | **0.01** |
|  |  | **Socialization** | **-5.14** | **-8.73** | **-1.54** | **0.01** |
|  |  | **Motor** | **-5.38** | **-9.15** | **-1.62** | **0.01** |
|  |  | **Composite score** | **-4.50** | **-7.54** | **-1.46** | **0.00** |
|  | ABC | **Irritability, agitation, crying** | **0.22** | **0.07** | **0.36** | **0.00** |
|  |  | **Lethargy, social withdrawal** | **0.28** | **0.13** | **0.43** | **0.00** |
|  |  | **Stereotypic behavior** | **0.28** | **0.10** | **0.47** | **0.00** |
|  |  | **Hyperactivity, non-compliance** | **0.19** | **0.06** | **0.32** | **0.00** |
|  |  | Inappropriate speech | -0.02 | -0.23 | 0.20 | 0.88 |
| DD | MSEL | Visual reception | -3.74 | -12.57 | 5.09 | 0.40 |
|  |  | **Fine motor** | **-5.14** | **-9.18** | **-1.10** | **0.01** |
|  |  | Receptive language | -6.41 | -13.94 | 1.12 | 0.09 |
|  |  | **Expressive language** | **-13.02** | **-22.02** | **-4.03** | **0.00** |
|  |  | Composite score | -7.40 | -14.78 | -0.02 | 0.05 |
|  | VABS | **Communication** | **-7.79** | **-14.43** | **-1.16** | **0.02** |
|  |  | Daily living | -4.40 | -10.72 | 1.91 | 0.17 |
|  |  | Socialization | -5.55 | -13.11 | 2.00 | 0.15 |
|  |  | **Motor** | **-8.48** | **-15.86** | **-1.09** | **0.02** |
|  |  | **Composite score** | **-6.45** | **-12.38** | **-0.52** | **0.03** |
|  | ABC | **Irritability, agitation, crying** | **0.22** | **0.07** | **0.36** | **0.00** |
|  |  | Lethargy, social withdrawal | 0.15 | -0.41 | 0.71 | 0.59 |
|  |  | **Stereotypic behavior** | **0.28** | **0.10** | **0.47** | **0.00** |
|  |  | Hyperactivity, non-compliance | 0.13 | -0.34 | 0.61 | 0.59 |
|  |  | Inappropriate speech | 0.46 | -0.09 | 1.02 | 0.10 |
| DS | MSEL | Visual reception | 3.70 | -3.30 | 10.70 | 0.29 |
|  |  | Fine motor | 1.17 | -4.68 | 7.02 | 0.69 |
|  |  | Receptive language | -1.52 | -10.10 | 7.05 | 0.72 |
|  |  | Expressive language | 2.74 | -4.91 | 10.38 | 0.48 |
|  |  | Composite score | 1.52 | -4.49 | 7.54 | 0.62 |
|  | VABS | Communication | 0.74 | -5.54 | 7.03 | 0.81 |
|  |  | Daily living | 5.55 | -2.31 | 13.41 | 0.16 |
|  |  | Socialization | 6.06 | -4.28 | 16.40 | 0.25 |
|  |  | Motor | 6.50 | -0.33 | 13.32 | 0.06 |
|  |  | Composite score | 4.91 | -1.37 | 11.18 | 0.12 |
|  | ABC | Irritability, agitation, crying | 0.28 | -0.16 | 0.71 | 0.22 |
|  |  | Lethargy, social withdrawal | 0.15 | -0.41 | 0.71 | 0.59 |
|  |  | Stereotypic behavior | 0.23 | -1.09 | 1.54 | 0.73 |
|  |  | Hyperactivity, non-compliance | 0.13 | -0.34 | 0.61 | 0.59 |
|  |  | Inappropriate speech | 0.38 | -1.25 | 2.01 | 0.65 |
| TD | MSEL | Visual reception | -2.08 | -10.30 | 6.14 | 0.62 |
|  |  | **Fine motor** | **-5.14** | **-9.18** | **-1.10** | **0.01** |
|  |  | Receptive language | -5.96 | -13.95 | 2.03 | 0.14 |
|  |  | **Expressive language** | **-9.38** | **-17.89** | **-0.87** | **0.03** |
|  |  | Composite score | -4.43 | -10.50 | 1.64 | 0.15 |
|  | VABS | Communication | -7.11 | -17.06 | 2.84 | 0.16 |
|  |  | Daily living | -4.62 | -13.68 | 4.45 | 0.32 |
|  |  | Socialization | -2.39 | -13.55 | 8.77 | 0.67 |
|  |  | Motor | 0.45 | -8.54 | 9.45 | 0.92 |
|  |  | Composite score | -3.38 | -10.96 | 4.21 | 0.38 |
|  | ABC | Irritability, agitation, crying | 0.21 | -0.42 | 0.83 | 0.51 |
|  |  | **Lethargy, social withdrawal** | **1.71** | **0.36** | **3.05** | **0.01** |
|  |  | Stereotypic behavior | 0.41 | -1.83 | 2.66 | 0.72 |
|  |  | Hyperactivity, non-compliance | 0.29 | -0.45 | 1.04 | 0.44 |
|  |  | Inappropriate speech | -0.30 | -1.51 | 0.91 | 0.62 |

**Supplementary Table 3**: Results of regression models predicting psychometric scores based on GI issues (continuous), stratified by sex. Models are adjusted for parental homeownership, maternal prenatal vitamin intake, and child’s year of birth. Significant associations in stratified models (p < 0.05) are shown in bold. P-values for heterogeneity are also presented.

|  |  |  | Females | | | |  | Males | | | | p-value  for  Heterogeneity |
| --- | --- | --- | --- | --- | --- | --- | --- | --- | --- | --- | --- | --- |
| Dx | Test | Subscale | Estimate | 95% CI | | p |  | Estimate | 95% CI | | p |  |
|  |  |  |  | Lower  Bound | Upper  Bound |  |  |  | Lower  Bound | Upper  Bound |  |  |
| ASD | MSEL | Visual reception | -3.63 | -7.66 | 0.40 | 0.08 |  | -0.55 | -2.72 | 1.62 | 0.62 | 0.14 |
|  |  | Fine motor | -2.44 | -6.20 | 1.31 | 0.20 |  | -1.40 | -3.12 | 0.32 | 0.11 | 0.50 |
|  |  | Visual reception | -2.48 | -6.74 | 1.78 | 0.25 |  | -0.23 | -2.49 | 2.03 | 0.84 | 0.29 |
|  |  | Expressive language | -2.90 | -6.83 | 1.04 | 0.15 |  | -0.91 | -3.02 | 1.20 | 0.40 | 0.32 |
|  |  | Composite score | -2.86 | -6.53 | 0.81 | 0.13 |  | -0.87 | -2.78 | 1.04 | 0.37 | 0.27 |
|  | VABS | Communication | -2.41 | -6.07 | 1.26 | 0.20 |  | **-1.94** | **-3.83** | **-0.06** | **0.04** | 0.77 |
|  |  | Daily living | **-3.20** | **-5.45** | **-0.95** | **0.01** |  | **-1.76** | **-2.95** | **-0.56** | **0.00** | 0.21 |
|  |  | Socialization | **-3.13** | **-5.91** | **-0.35** | **0.03** |  | **-1.88** | **-3.46** | **-0.29** | **0.02** | 0.40 |
|  |  | Motor | **-4.06** | **-7.37** | **-0.76** | **0.02** |  | **-2.25** | **-3.84** | **-0.66** | **0.01** | 0.24 |
|  |  | Composite score | **-2.91** | **-5.41** | **-0.40** | **0.02** |  | **-1.96** | **-3.28** | **-0.64** | **0.00** | 0.43 |
|  | ABC | Irritability, agitation, crying | 0.10 | -0.02 | 0.22 | 0.09 |  | **0.17** | **0.11** | **0.23** | **<.000** | 0.30 |
|  |  | Lethargy, social withdrawal | **0.14** | **0.03** | **0.26** | **0.01** |  | **0.16** | **0.09** | **0.22** | **<.0001** | 0.81 |
|  |  | Stereotypic behavior | **0.21** | **0.06** | **0.36** | **0.01** |  | **0.17** | **0.09** | **0.24** | **<.0001** | 0.30 |
|  |  | Hyperactivity, non-compliance | **0.14** | **0.05** | **0.24** | **0.00** |  | **0.12** | **0.06** | **0.17** | **<.0001** | 0.68 |
|  |  | Inappropriate speech | 0.06 | -0.07 | 0.20 | 0.36 |  | 0.05 | -0.05 | 0.14 | 0.34 | 0.80 |
| DD | MSEL | Visual reception | 0.10 | -5.84 | 6.04 | 0.97 |  | -4.24 | -9.22 | 0.73 | 0.09 | 0.22 |
|  |  | Fine motor | -0.15 | -5.60 | 5.30 | 0.96 |  | **-5.50** | **-9.60** | **-1.39** | **0.01** | 0.10 |
|  |  | Visual reception | 1.48 | -3.68 | 6.64 | 0.57 |  | **-5.25** | **-9.47** | **-1.03** | **0.02** | 0.04 |
|  |  | Expressive language | -0.66 | -7.04 | 5.72 | 0.84 |  | **-6.77** | **-11.57** | **-1.97** | **0.01** | 0.09 |
|  |  | Composite score | 0.19 | -4.93 | 5.31 | 0.94 |  | **-5.44** | **-9.48** | **-1.40** | **0.01** | 0.07 |
|  | VABS | Communication | -0.63 | -5.09 | 3.84 | 0.78 |  | **-6.26** | **-9.93** | **-2.60** | **0.00** | 0.04 |
|  |  | Daily living | -0.10 | -4.02 | 3.82 | 0.96 |  | **-5.36** | **-9.00** | **-1.72** | **0.00** | 0.04 |
|  |  | Socialization | -0.24 | -5.29 | 4.81 | 0.92 |  | -3.95 | -8.27 | 0.37 | 0.07 | 0.25 |
|  |  | Motor | 0.58 | -3.85 | 5.01 | 0.80 |  | **-6.44** | **-10.80** | **-2.08** | **0.00** | 0.03 |
|  |  | Composite score | -0.01 | -3.69 | 3.67 | 0.99 |  | **-5.54** | **-8.93** | **-2.15** | **0.00** | 0.03 |
|  | ABC | Irritability, agitation, crying | -0.02 | -0.31 | 0.27 | 0.91 |  | 0.16 | -0.09 | 0.41 | 0.20 | 0.33 |
|  |  | Lethargy, social withdrawal | **0.40** | **0.07** | **0.74** | **0.02** |  | 0.04 | -0.28 | 0.35 | 0.82 | 0.11 |
|  |  | Stereotypic behavior | 0.35 | -0.20 | 0.91 | 0.21 |  | 0.31 | -0.06 | 0.69 | 0.10 | 0.33 |
|  |  | Hyperactivity, non-compliance | 0.11 | -0.21 | 0.43 | 0.50 |  | 0.02 | -0.25 | 0.28 | 0.91 | 0.62 |
|  |  | Inappropriate speech | 0.19 | -0.16 | 0.54 | 0.29 |  | 0.26 | -0.06 | 0.58 | 0.11 | 0.75 |
| DS | MSEL | Visual reception | 1.51 | -7.45 | 10.48 | 0.73 |  | 4.53 | -0.56 | 9.61 | 0.08 | 0.52 |
|  |  | Fine motor | 0.28 | -6.74 | 7.30 | 0.94 |  | 0.79 | -3.78 | 5.36 | 0.73 | 0.84 |
|  |  | Visual reception | -2.66 | -12.68 | 7.35 | 0.59 |  | 3.41 | -3.42 | 10.24 | 0.32 | 0.29 |
|  |  | Expressive language | 3.62 | -5.03 | 12.26 | 0.40 |  | 2.08 | -3.98 | 8.15 | 0.49 | 0.75 |
|  |  | Composite score | 0.69 | -6.64 | 8.01 | 0.85 |  | 2.70 | -1.83 | 7.23 | 0.23 | 0.61 |
|  | VABS | Communication | 1.31 | -6.34 | 8.97 | 0.73 |  | -0.12 | -5.05 | 4.81 | 0.96 | 0.75 |
|  |  | Daily living | 2.31 | -6.06 | 10.68 | 0.58 |  | 1.10 | -5.77 | 7.97 | 0.75 | 0.87 |
|  |  | Socialization | -0.39 | -12.55 | 11.77 | 0.95 |  | 1.57 | -6.51 | 9.65 | 0.70 | 0.78 |
|  |  | Motor | 3.39 | -3.20 | 9.99 | 0.30 |  | -0.59 | -7.16 | 5.99 | 0.86 | 0.40 |
|  |  | Composite score | 1.76 | -5.23 | 8.75 | 0.61 |  | 0.58 | -4.82 | 5.97 | 0.83 | 0.80 |
|  | ABC | Irritability, agitation, crying | 0.15 | -0.61 | 0.91 | 0.71 |  | 0.14 | -0.50 | 0.79 | 0.66 | 0.71 |
|  |  | Lethargy, social withdrawal | -0.02 | -1.98 | 1.94 | 0.98 |  | 0.72 | -0.89 | 2.33 | 0.38 | 0.71 |
|  |  | Stereotypic behavior | -0.65 | -2.55 | 1.25 | 0.50 |  | -0.08 | -1.32 | 1.17 | 0.90 | 0.71 |
|  |  | Hyperactivity, non-compliance | -0.25 | -0.97 | 0.48 | 0.50 |  | 0.34 | -0.43 | 1.11 | 0.39 | 0.56 |
|  |  | Inappropriate speech | 1.21 | 0.00 | 2.42 | 0.05 |  | -0.78 | -2.78 | 1.21 | 0.44 | 0.07 |
| TD | MSEL | Visual reception | -1.61 | -9.47 | 6.26 | 0.69 |  | -2.28 | -6.39 | 1.83 | 0.28 | 0.65 |
|  |  | Fine motor | 1.32 | -4.67 | 7.31 | 0.66 |  | -0.18 | -3.61 | 3.25 | 0.92 | 0.64 |
|  |  | Visual reception | 0.01 | -6.15 | 6.16 | 1.00 |  | -3.25 | -7.43 | 0.93 | 0.13 | 0.40 |
|  |  | Expressive language | -0.39 | -8.14 | 7.36 | 0.92 |  | -3.36 | -7.72 | 1.01 | 0.13 | 0.45 |
|  |  | Composite score | -0.17 | -5.52 | 5.19 | 0.95 |  | -2.27 | -5.35 | 0.82 | 0.15 | 0.41 |
|  | VABS | Communication | -2.44 | -11.46 | 6.59 | 0.59 |  | -3.24 | -8.31 | 1.83 | 0.21 | 0.80 |
|  |  | Daily living | -1.19 | -9.68 | 7.31 | 0.78 |  | -2.80 | -7.35 | 1.74 | 0.23 | 0.68 |
|  |  | Socialization | -3.37 | -13.11 | 6.36 | 0.49 |  | -1.63 | -7.35 | 4.08 | 0.57 | 0.69 |
|  |  | Motor | 1.15 | -7.74 | 10.04 | 0.80 |  | 1.15 | -3.36 | 5.65 | 0.62 | 0.87 |
|  |  | Composite score | -1.76 | -8.87 | 5.35 | 0.62 |  | -1.68 | -5.48 | 2.12 | 0.39 | 0.97 |
|  | ABC | Irritability, agitation, crying | -0.03 | -0.61 | 0.54 | 0.91 |  | 0.35 | -0.01 | 0.70 | 0.06 | 0.12 |
|  |  | Lethargy, social withdrawal | 0.59 | -0.42 | 1.60 | 0.26 |  | **0.87** | **0.11** | **1.63** | **0.03** | 0.72 |
|  |  | Stereotypic behavior | 0.44 | -2.54 | 3.42 | 0.77 |  | **1.61** | **0.25** | **2.98** | **0.02** | 0.12 |
|  |  | Hyperactivity, non-compliance | 0.01 | -0.61 | 0.62 | 0.98 |  | 0.34 | -0.13 | 0.82 | 0.16 | 0.19 |
|  |  | Inappropriate speech | -0.09 | -0.97 | 0.78 | 0.83 |  | 0.31 | -0.21 | 0.84 | 0.24 | 0.70 |

**Supplementary Fig:** Directed acyclic graph (DAG) used to identify potential confounders of the relationship between GI issues and psychometric scores.

**
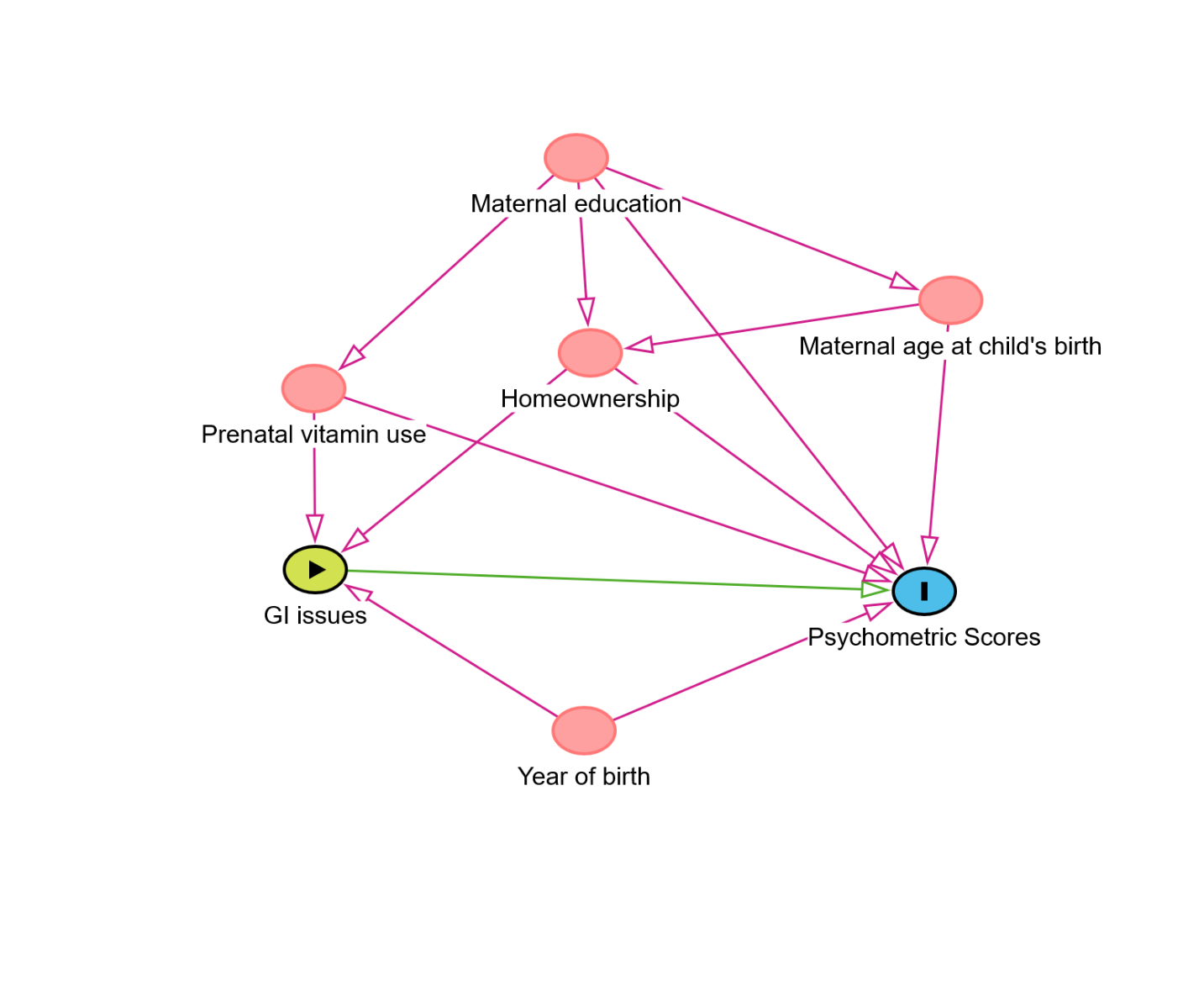
**

*Home ownership is a proxy for family wealth.
